# Mutationmapper: A Tool to Aid the Mapping of Protein Mutation Data

**DOI:** 10.1371/journal.pone.0071711

**Published:** 2013-08-09

**Authors:** Shabana Vohra, Philip C. Biggin

**Affiliations:** 1 Structural Bioinformatics and Computational Biochemistry, University of Oxford, Oxford, United Kingdom; 2 Oxford Centre for Integrative Systems Biology, Department of Biochemistry, Oxford, United Kingdom; CSIR-Institute of Microbial Technology, India

## Abstract

There has been a rapid increase in the amount of mutational data due to, amongst other things, an increase in single nucleotide polymorphism (SNP) data and the use of site-directed mutagenesis as a tool to help dissect out functional properties of proteins. Many manually curated databases have been developed to index point mutations but they are not sustainable with the ever-increasing volume of scientific literature. There have been considerable efforts in the automatic extraction of mutation specific information from raw text involving use of various text-mining approaches. However, one of the key problems is to link these mutations with its associated protein and to present this data in such a way that researchers can immediately contextualize it within a structurally related family of proteins. To aid this process, we have developed an application called MutationMapper. Point mutations are extracted from abstracts and are validated against protein sequences in Uniprot as far as possible. Our methodology differs in a fundamental way from the usual text-mining approach. Rather than start with abstracts, we start with protein sequences, which facilitates greatly the process of validating a potential point mutation identified in an abstract. The results are displayed as mutations mapped on to the protein sequence or a multiple sequence alignment. The latter enables one to readily pick up mutations performed at equivalent positions in related proteins. We demonstrate the use of MutationMapper against several examples including a single sequence and multiple sequence alignments. The application is available as a web-service at http://mutationmapper.bioch.ox.ac.uk.

## Introduction

The amount of pharmacological and physiological literature pertaining to proteins has increased enormously over recent years [1]. Indeed, this is part of the so-called data-deluge and poses several problems for researchers in terms of dealing with the huge volumes of data [2]. As such it is becoming increasingly difficult, if not impossible, for researchers to keep up to date with the literature that may be relevant, either directly or indirectly, to their particular area of research. In order to address these issues, much effort has gone into the development of text-mining methodologies to facilitate the extraction of data directly from the literature [2]–[5]. Such methods have the potential not only to aid researchers keep pace with the literature but also to discover novel relationships between aspects of proteins that may be important. Equally as important as the data extraction itself is the presentation and usability of the data that is mined.

A key tool in the arsenal of the molecular biologist to study function is site-directed mutagenesis (SDM). The effect of a single mutation can have a range of effects on protein function; from completely non-functional to a slight change in affinity for a known ligand. Additionally, point mutations are known to be important in the appearance of many disease states. In addition to mutations found/made for a particular protein, there is much greater potential for scientific discovery if one can utilize information about mutations found at similar positions from related proteins (however that may be defined). Knowledge about the impact of all mutations at a conserved position within a series of related proteins would help our understanding of all of those proteins and may reveal undiscovered relationships or suggest structurally or functionally important positions.

Traditional keyword information searching performed in PubMed is inefficient at retrieving mutational data; typically one retrieves too many abstracts to be useful. The alternatives to automatic retrieval are manually curated systems such as PMD [6], but this requires huge effort that is probably not sustainable in the longer term. Several algorithms [7]–[9] have been developed to extract point mutations from the literature but the challenge is to relate the mutation to the associated protein. To that end we have developed an application called MutationMapper that uses an integrated pipeline to extract point mutations from published text and map them on to the associated protein. The process of identifying a particular protein in the literature is a non-trivial task because there is no standard way of naming them [10], [11]. The most common approach utilized in workflows in text-mining applications is to search through a large amount of text-based data. The first step before any mining can begin is to limit the size of the data. This has traditionally been achieved by the use of indexing terms or keywords. In the case of medically-related subjects, the medical subject heading (MESH) terms are often used.

To help circumvent some of these problems we adopted a different approach. Rather than depend directly on MESH terms or keywords, the input is a sequence (or multiple sequence alignment) identified by the Uniprot ID or Accession code. This information is used to obtain information regarding protein names and synonyms from Uniprot which is subsequently used to query PubMed. Although the search terms will be similar to a conventional search, we have the additional knowledge at this point as to which proteins we are most interested in. Starting from this perspective also has the advantage that the user can supply their own multiple sequence alignment and make their own judgment about the value of the data that comes back from the query. Furthermore, it facilitates the discovery of new relationships between sequences that might not normally be compared to each other. Because we leave the final interpretation of the data to the user, the issue of data accuracy, although important, becomes less critical to the usefulness of the application.

## Materials and Methods

**Figure 1:**
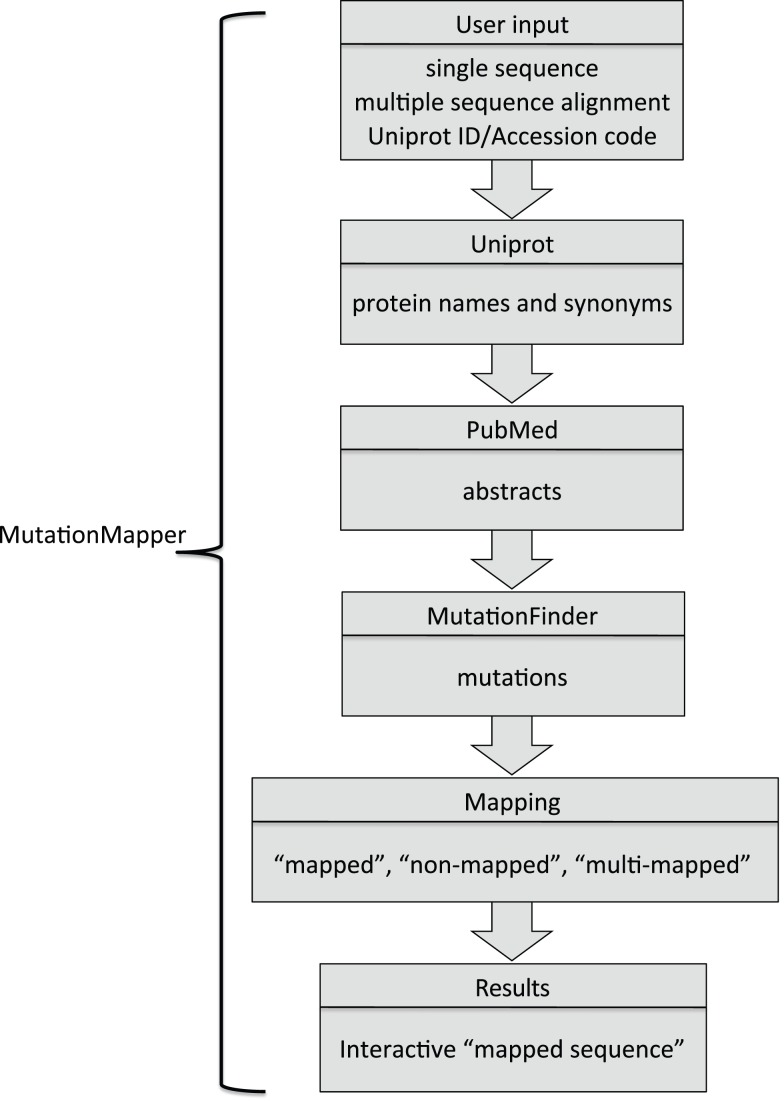
Schematic flow chart of the process used in MutationMapper. The starting point is a single sequence or multiple sequence alignment with the Uniprot ID or Accession code as the identifier. The identifier is used to query Uniprot to retrieve protein names, gene names and synonyms which are then used to retrieve abstracts from PubMed. Abstracts are converted to raw text and then the program MutationFinder [8] is used to extract possible mutations. These mutations are then mapped back to the protein sequence(s) with three possible outcomes: i) mapped, ii) non-mapped and iii) multi-mapped. Only mapped and multi-mapped results are highlighted on the sequence (or multiple sequence alignment) presented back to the user.

As mentioned above, the design philosophy for MutationMapper differs from previous text-mining applications. The starting point is the protein sequence(s) rather than the literature. The user can provide a single sequence for which mutational data is requested for, or the user can provide a multiple sequence alignment (MSA) for which mutations will be searched for across all the proteins and overlaid on top of the given alignment. Provision of an alignment is only needed in order to visualize mutations at equivalent positions. It does not effect retrieval or mapping in any way and is not necessary in order to obtain mutational data for a single protein sequence. The overall workflow is illustrated in Figure 1.

The input to MutationMapper, via a web-interface, is a Uniprot ID or a single protein sequence or an MSA in FASTA format with Uniprot Id or Accession code as the sequence identifier. The sequence identifier is used to retrieve protein information from Uniprot, including protein names, gene names, commonly recommended names and synonyms. This information is used to query PubMed and retrieve abstracts. Once abstracts are retrieved and converted to raw text, the next stage is to extract possible mutation data. For that purpose we used the freely available MutationFinder program [7]. It splits text at the sentence level and applies sets of regular expressions to identify and extract mutations. It reports the mutations in wNm format where ‘w’ is the wild type residue, ‘m’ is the mutated residue and ‘N’ is the residue position.

The critical step is to map a particular mutation that is found within the text to the correct protein. In other words we need to validate the position of the mutation with respect to a given protein. Essentially, the wild type residue is looked for within the amino acid sequence (as given by Uniprot). If a match is found, the position is marked as “mapped”. If the algorithm fails to identify any protein name or is not able to map to the identified protein the mutation is labeled as “non-mapped”. If a mutation is mapped on more than one protein sequence than the information is processed further to map it down to one sequence [12]. The algorithm looks for protein expressions (i.e. sections of text that pertain to proteins) in one sentence above and one below, including the sentence with the mutation expression in, and if it still maps on more than one protein it is marked as “multi-mapped”. The algorithm also maps the mutations reported in Uniprot entries [13], [14]. These mutations are natural variants of the proteins and the mutagenesis data. The natural variants include polymorphisms, variations between strains, isolates or cultivars, disease-associated mutations and RNA editing events. Information on disease-associated mutations is however, mostly restricted to human proteins.

**Figure 2:**
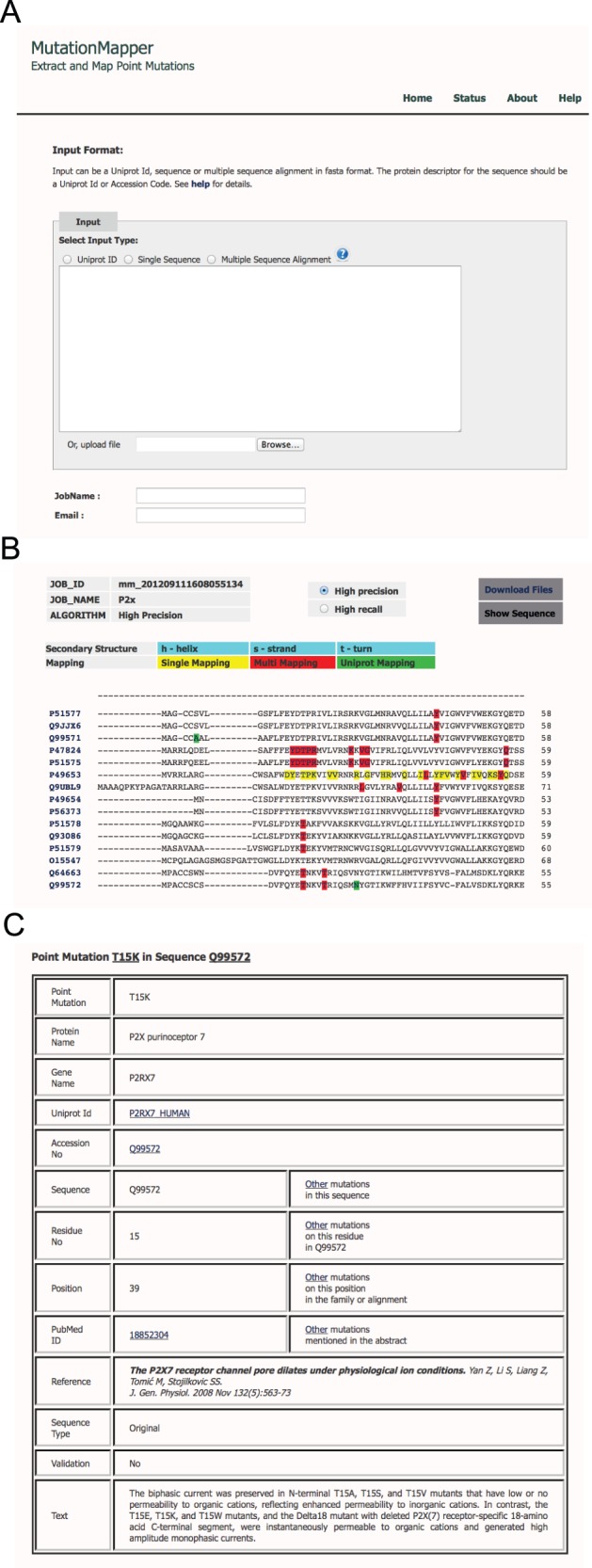
Example screenshots from (A) Starting submission screen, (B) multiple-sequence alignment for P2X proteins and (C) detailed information screen from a mutation found for the P2X7 protein.

The results are presented as highlighted positions on the sequence or the alignment provided by the user (see Figure 2 for example screenshots), thus providing an easy way to instantly see whether mutation data exists at a particular position within the protein or for similar proteins at similar positions. One can browse all the mutations in a particular sequence or at a particular position or a particular abstract. The relevant text pertaining to the mutation can also be viewed by the user.

## Results and Discussion

### Performance Evaluation

Clearly, such a tool is only useful if the user has knowledge of how well the mutation-mining process performs in terms of retrieving real mutation data that pertains to a protein sequence and not just matching phrases that might be interpreted as such.

Within text-mining and related fields, one can make use of additional indicators such as recall, precision, accuracy and the F-measure. These can be defined in terms of true positive (tp), false positive (fp), false negative (fn) and true negative (tn) as follows:-
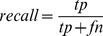
(1)

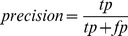
(2)

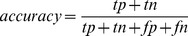
(3)

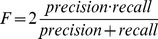
(4)


We evaluated the efficiency of MutationMapper using various single sequences and a few multiple sequence alignments as test cases. The performance was measured in terms of recall, precision, accuracy and F-measure. All the abstracts with mutations determined by MutationFinder [8] were manually curated to validate the mapping process in order to evaluate the performance of the method.

### Single Sequence

The performance of MutationMapper was first evaluated against test cases involving a single sequence. We show here the results for three examples; the human phosphatase and tensin homologue (PTEN) protein [15], the human glycophorin protein [16] and the chemotaxis protein CheY from *E. coli* [17]. The input into the MutationMapper server was a Uniprot ID for that protein or a single sequence with a Uniprot ID or Accession code as the descriptor. Table 1 summarizes the mutations retrieved from abstracts for these proteins. Recall was 100% in all three proteins. The precision was ∼98% for PTEN_HUMAN and CHEY_ECOLI and ∼85% for GLPA_HUMAN. The accuracy and F-measure was high (greater than 95%) in all three test cases (see Figure S1 in File S1).

**Table 1:** Information retrieval and mutation extraction in three test cases.

Sequence	Keywords*	Abstractsretrieved	Abstracts with mutations	Number of mutations
PTEN_HUMAN	“PTEN”, “PHOSPHATASE AND TENSIN HOMOLOG”, “MMAC1”, “TEP1”	6001	297	435
GLPA_HUMAN	“GLYCOPHORIN-A”, “MN SIALOGLYCOPROTEIN”,“SIALOGLYCOPROTEIN ALPHA”, “GYPA”, “GPA”	8837	95	183
CHEY_ECOLI	“CHEMOTAXIS PROTEIN CHEY”,”CHEY”	933	51	103

* These are the keywords that MutationMapper automatically extracted from Uniprot and used to search PubMed.

In PTEN_HUMAN, out of 6001 abstracts retrieved, 297 were found to contain information concerning mutations with a total of 435 possible mutations. Out of the 435 mutations, we were able to map 125 mutations. Thus, 310 were not mapped. There were 121 true positives (i.e. mutations mapped back to the correct protein) and it mapped all the true positives with 100% recall. All the non-mapped mutations were true negatives, as they were not reported on PTEN_HUMAN, but for other proteins or other factors (see below). The false positives (four in total) were mutations mapped on the wrong protein. We had no false negatives. There was an accuracy of 99% and an F-measure of 98.3%. The number of true negatives was high and this reflects a number of issues surrounding the identification of mutations from abstracts: 1) A large proportion were gene mutations rather than protein mutations (e.g. PubMed ID (PMID) 19665071 where a gene has a “point mutation in exon 5 (A536G)”, PMID 16009891 which has a “major pathogenic effect at mRNA level for the mutant C1366T” and PMID 11597326 which has “an intronic variant of p53–G13964C”); 2) some were on another protein that was not the subject of the query. For example, PMID 16160475, where “six different beta-catenin mutations were found in 7 of 13 cases (54%) (G34E, G34V, S33C, D32Y, S33F, D32A) however, no mutations of the PTEN or K-ras genes were identified”. In this example the mutation was performed on beta-catenin and not PTEN even though the sequence being queried against was the human PTEN sequence; 3) some were the names of cell lines. For example, “T47D cells” or “human glioblastoma cell line T98G” and 4) in some cases there was no specific protein mentioned. Similar performance was observed for other test cases (data not shown). In the context of our methodology presented here, these true-negatives are not a particular problem as we are able to trap them, but it does highlight the difficulty in being able to reliably identify genuine mutations with text-mining alone. In fact this exemplifies the key challenge because mutations are often not described in accordance with any standard nomenclature. For example, Wei et al. [18] recently reported that less than 25% of mutations in their corpus of abstracts used to evaluate tmVar were reported using standard nomenclature. These authors also reported similar problems to those identified above and others including the identification of missense mutations such as V125X. Another example we identified is where standard mutation nomenclature is appended by some other text such as “Y1472F-KI”, where the KI means “knock in” mice [19].

The use of a short-hand nomenclature based on motifs is quite common in the literature and creates difficulties for a solution that attempts to be generic. Typically, authors then refer to a position in a specific motif. For example, in NMDA receptors there is an invariant SYTANLAAF motif that has been investigated by mutational analysis. For convenience, these positions are sometimes referred to by their position with respect to the first S of this motif, so a mutation of the second tyrosine to phenylalanine might be described as Y2F (e.g. PMID 22891278 [20]) rather than the full-length or mature protein numbering. In fact this problem is possibly more widespread than first thought; a similar “shorthand” numbering scheme has been used previously in the cys-loop family of receptors to describe positions along the pore-lining M2 helices [21] (see for example PMID 15213309 [22]). As well as those cited above, a further example would be the G-protein Coupled Receptors (GPCRs) where the so-called Ballesteros and Weinstein numbering scheme [23] is commonly used rather than the position in the full length of mature sequences. Thus, one must temper user expectations of what is possible within text mining and in some cases it may well be more desirable to use an “entity-specific” method such as those created for ABC transporters [24] and Fabry disease [25].

The false positives for the PTEN_HUMAN sequence are due to 1) mutations being reported on another protein but which map onto the query due to a coincidental occurrence that the amino acid found at that position was identical in both proteins and; 2) mutations being reported on a closely related sequence. In glycophorin, we observed mutations reported on type B but since GLPA and GLPB are highly similar, it was mapped on type A (which was the subject of our query). There were no false negatives in the above examples. One of the reasons for false negatives is when the commonly used protein names in published text are not retrieved from Uniprot, hence the algorithm fails to identify the protein names in the abstract and is consequently unable to map it.

### Multiple Sequence Alignment

Although mapping mutations to single sequences automatically is useful in its own right, the real power of the methodology is to map mutations to an alignment so that users can readily visualize data at common positions. We evaluated the performance on multiple sequence alignments using a set of P2X receptors and ionotropic glutamate receptors (iGluR) from rat as two distinct test cases (see Figures S2 and S3 in File S1). The sequence identifiers within the multiple sequence alignments are used to query Uniprot for protein names that are then used to query PubMed as described for single sequences (see Figure 1 and Materials and Methods).

For P2X receptors, 18070 abstracts were retrieved, 920 mutations were reported in 336 abstracts. Out of the 920 mutations, we were able to map 486 mutations (311 mutations mapped more than once) and 434 were not mapped. Out of 486 mutations mapped, there were 482 true positives and 4 false positives giving a precision of 99% (Figure S2 in File S1). All the non-mapped mutations were true negatives with no false negatives, so the recall was 100%. The number of multi-mapped mutations very high in this case but they were mapped correctly. The abstract data contained sequences from P2RX1, P2RX2, P2RX3, P2RX4, P2RX5 P2RX6 and P2RX7 receptors from rat, mouse and human. As the sequence similarity between these species is high, the chances of the same residue being found at the same position is also high, thus giving a high multi-mapped rate.

In the case of iGluRs, 45339 abstracts were retrieved and 1254 mutations were reported in 650 abstracts. We were able to map 239 mutations (with 13 mutations mapped more than once) and 1015 were not mapped. Out of 239 mutations mapped, there were 206 true positives and 33 false positives giving a precision of 86%. Out of 1015 non-mapped mutations, there were 910 true negatives and 105 false negatives, so the recall was 66% (Figure S3 in File S1). Since the recall was quite low in this case, we decided to implement variations to the algorithm to help improve this aspect.

We call the variations "high precision" and "high recall". We imagine that a small number of false positives could be tolerated by users, but at the same time, users would want to be sure that as many mutations as possible were mapped (recall). The trade off between these two options cannot readily be predicted and thus the simplest solution is to let the user explore the options for each individual case. Thus, we give the user the ability to browse the results of both algorithms. In high precision mode, the algorithm does an exact match for the protein expression. For example in the case of the GRIK5_RAT sequence, one of the protein expressions retrieved from Uniprot is “Glutamate receptor, ionotropic kainate 5″. Only if all of the words in the expression are found is the protein name used for mapping. In high recall mode, only two or more words are required from the expression for the protein name to be mapped. Using the high precision variant on the rat iGluR MSA gave a recall of 70% and a precision of 90%. In high recall mode, the recall was 99% and the precision was 33% (Figure S4 in File S1).

### The Issue of Multiple-mapping Events

The original objective was to accurately map a mutation mentioned in the text back to a single protein sequence, thus giving the user confidence that the mutation discussed really does correspond to the protein of interest. However, in certain scenarios, and as a consequence of the methodology, it may not be possible to map the mutation to a single protein sequence. For example if there are two closely related sequences in the alignment and there is a mutation performed at a certain position in one of the sequences and that position has the same amino acid at that position in two (or more) sequences, then the method cannot disambiguate them. One would expect this to occur more frequently the higher the percentage identity. However, in the context of the envisaged usage (i.e. looking for mutations performed across related proteins), we argue that this is in fact not a problem, because ultimately it is the reporting of a mutation at that position in the alignment that is of interest.

### User Input Expressions

To aid retrieval we also provide the option of including protein name expressions. As well as helping with ambiguity, it can also be useful if Uniprot does not include protein expressions that are commonly found in the published text. When a user runs a job, they are able to download the protein expression file that was used to search PubMed. The user can then modify the file to include additional expressions and rerun the job with those new expressions.

An illustrative example is the case of the NMDZ1_RAT entry from Uniprot. The expression "NR1" is commonly used in the literature but this is not present as an expression in Uniprot. When this expression is included manually, the number of abstracts retrieved can be significantly increased (Table 2) with a concomitant rise in true positives (Figure S5 in File S1).

**Table 2:** Information retrieval in NMDZ1_RAT with user input expressions.

	MM Expressions	User Input Expressions
**Keywords**	"GLUTAMATE NMDA RECEPTOR SUBUNIT ZETA1","NMETHYLDASPARTATE RECEPTOR SUBUNIT NR1","NMDR1", "GRIN1", "NMDAR1"	"GLUTAMATE NMDA RECEPTOR SUBUNIT ZETA1", "NMETHYLDASPARTATE RECEPTOR SUBUNIT NR1", "NMDR1", "GRIN1", "NMDAR1", **"NR1"**
**Abstracts retrieved**	2179	3178
**Abstracts with mutations**	77	103
**Number of Mutations**	143	218

Another example is PEPT1, where the single Uniprot protein expression “Macrophage oligopeptide transporter PEPT1” retrieves only one abstract when queried against PubMed. But if we add an additional expression consisting of just “PEPT1”, 630 abstracts with 60 mutations are retrieved. Hence the addition of a user-informed expression can be particularly effective in such cases.

Some protein expressions are similar to commonly used words or may only be a few letters long. The result is that the number of irrelevant abstracts retrieved is too large to be manageable. For example, for the matrix protein from influenza B (Uniprot entry BM2_INBMP), the gene name is ‘M’. This results in an enormous number of abstracts most of which are irrelevant. To avoid such problems and to increase the efficiency of the searches, we apply a filter. Words with two or less characters are ignored. Words with three characters are checked for the presence of a number. If a number is found, that string is used, otherwise it is discarded. Words with greater than three characters are used in the search.

### Mining for Mutations from Full-text

A commonly perceived view is that the most complete retrieval of mutation data can only be accomplished by searching the full text of articles. The retrieval of full-text presents additional problems [26], not least of which is the availability of that text online. Nevertheless, we were interested to see to what extent the inclusion of full text articles would have on the performance of MutationMapper. Thus, we retrieved as many full-text articles pertaining to ionotropic glutamate receptors as our institutional access allowed. Out of 3170 articles retrieved, 1511 mutations were reported in 376 articles, 579 were single-mapped whereas 405 were multi-mapped. However we found that the performance in terms of accuracy was substantially reduced (as assessed by manual inspection). The reduction in accuracy was rather unexpected. However, on manual reading of the extracted texts there appears to be more scope for ambiguity. Another potential problem is when mutations are referred to in figure legends, which tend to be written as briefly as possible, making association with the correct protein difficult. An even worse scenario is when mutations are referred to within figures (e.g. Mejias et al. [27]), which is almost impossible to accurately extract and map back to effectively. We suggest that this probably reflects author’s tendency to discuss other proteins within the more general discussions of a complete paper rather than the more focused confines of the abstract as has been discussed before [28].

### Conclusions

We have developed a pipeline that can aid researchers in discovering literature pertaining to mutation data. The novel aspect of this pipeline is that it allows mutations to be analyzed in the context of a sequence alignment. Quite often a mutation is performed on one member of a protein family that might have structural meaning in the context of another. The searches against full-text were less informative than against abstracts only. Searches are likely to improve as textual data becomes more annotated. Although there is progress in this area [29]–[32], this is a separate and complex issue in its own right. Nevertheless, such annotation should improve the prospects for greater accuracy. Although there is still a long way to go with accuracy concerning mutation mining, the system here provides a quick first pass method to ascertain mutational data that might be of relevance. The methodology is available as a web service at http://mutationmapper.bioch.ox.ac.uk.

## Supporting Information

File S1 **A single file containing five supporting information figures.**
(PDF)
