# Supplementary material for: Mutationmapper: A Tool to Aid the Mapping of Protein Mutation Data
Source: PLoS One. 2013 Aug 9;8(8):e71711. doi: 10.1371/journal.pone.0071711 (PMC3739722; doi:10.1371/journal.pone.0071711)
Supplement: File S1 — A single file containing five supporting information figures. (PDF) [file pone.0071711.s001.pdf]

Supporting Information for Vohra and Biggin.

In this file we present summary histograms for the statistical measures for the test case and the multiple sequence alignments used for PTEN and ionotropic glutamate receptors.

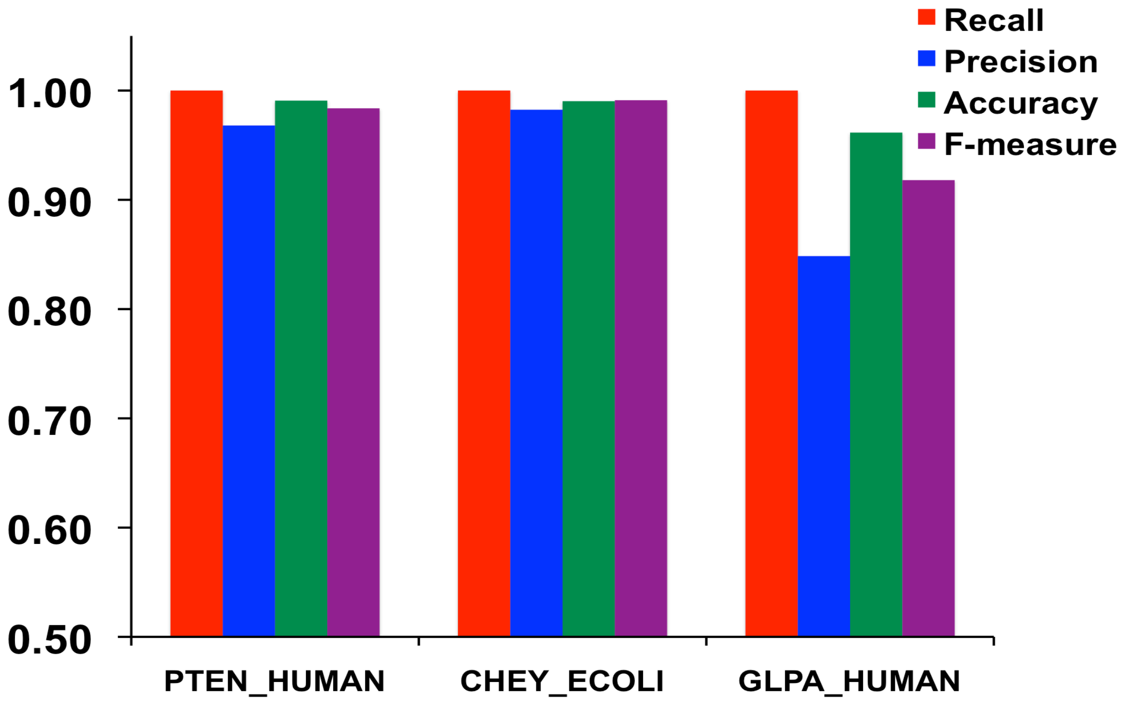

Figure S1. Evaluation of performance for MutationMapper on single sequence showing recall, precision, accuracy and F-measure in PTEN_HUMAN, CHEY_ECOLI, GLPA_HUMAN. There is high performance in all three test cases.

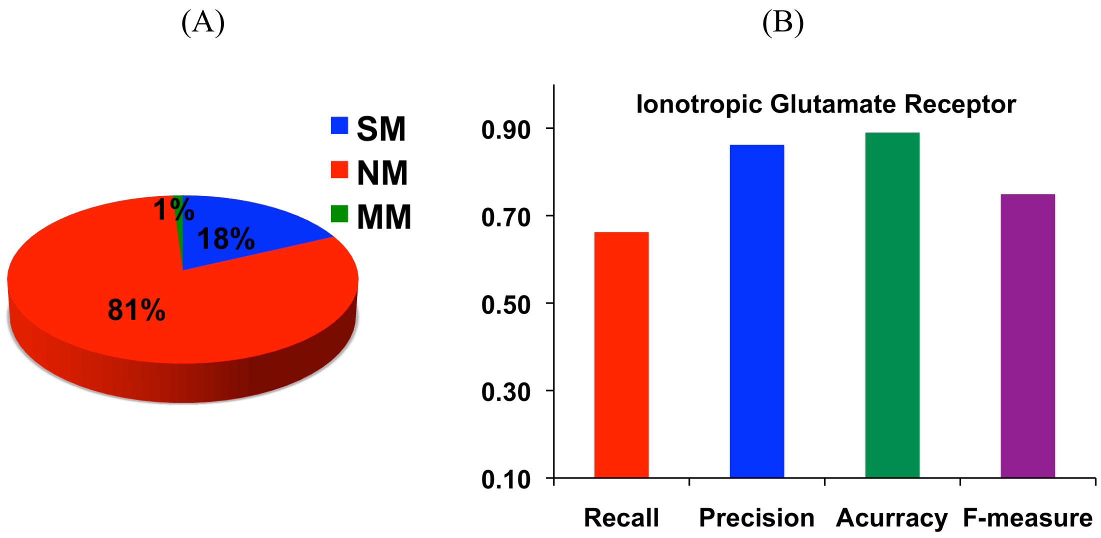

Figure S2. Performance Evaluation for MutationMapper in Ionotropic Glutamate Receptors (MSA) using High Precision (A) Percentage of mutations mapped once (blue), mapped more than once (green) and mutations not mapped (red). (B) Recall, precision, accuracy and F-measure statistics.

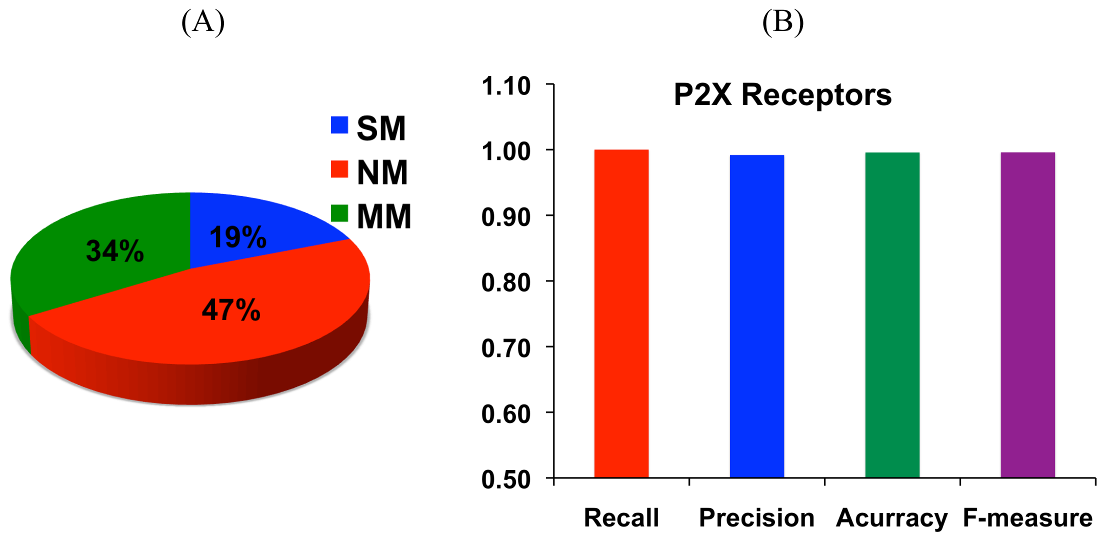

Figure S3. Performance evaluation for MutationMapper in P2X Receptors (MSA) using "High Precision" mode. (A) shows the percentage of mutations mapped once (blue), more than once (green) and mutations not mapped (red). (B) shows the recall, precision, accuracy and F-measure statistics.

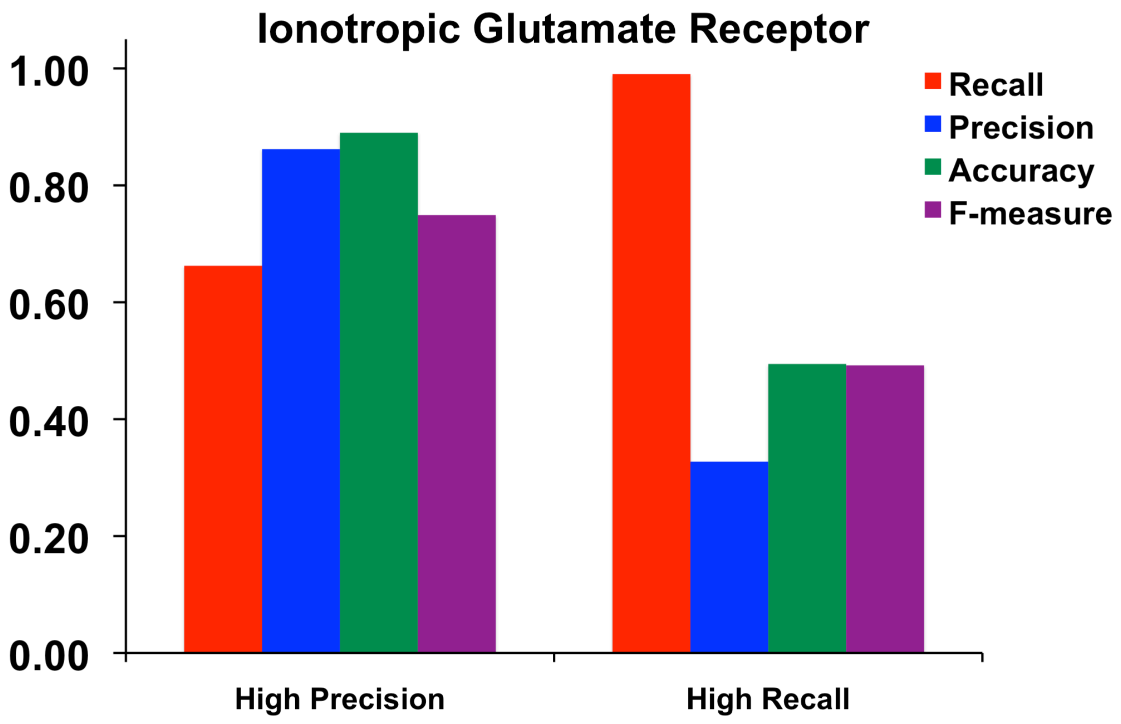

Figure S4. Performance comparisons of “High Precision” and “High Recall” modes in the Ionotropic glutamate receptor test cases.

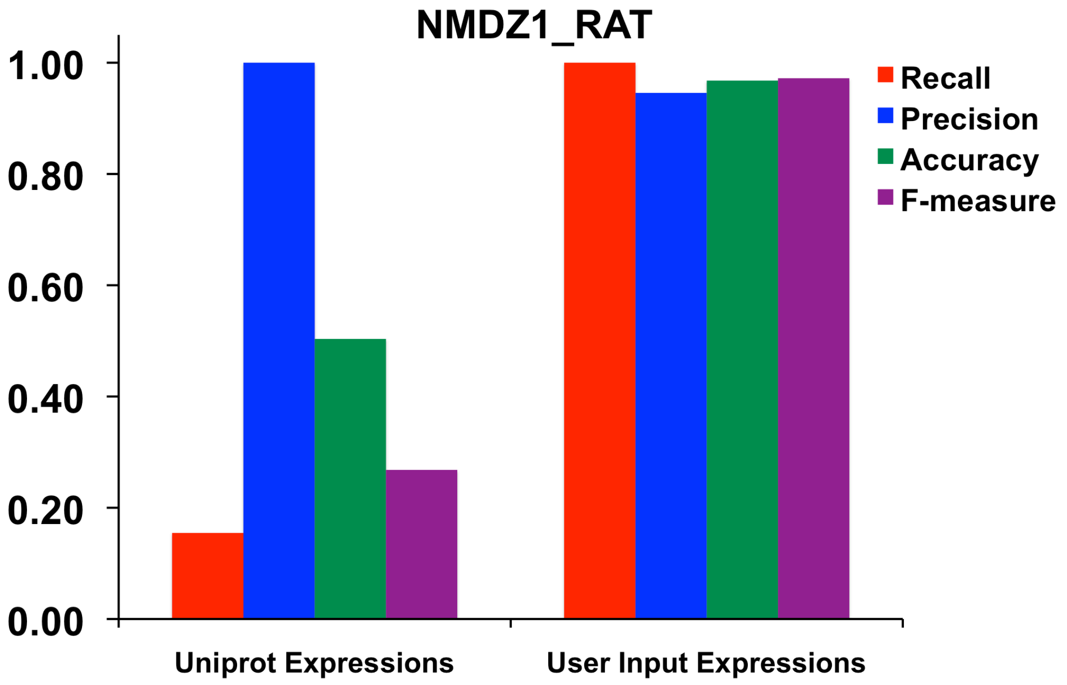

Figure S5. Performance can be improved with user input expression. There were no false negatives in this test case.
